# Intragenic tandem repeat variation between *Legionella pneumophila *strains

**DOI:** 10.1186/1471-2180-8-218

**Published:** 2008-12-10

**Authors:** David A Coil, Liesbeth Vandersmissen, Christophe Ginevra, Sophie Jarraud, Elke Lammertyn, Jozef Anné

**Affiliations:** 1 Laboratory of Bacteriology, Rega Institute for Medical Research, Katholieke Universiteit Leuven, Minderbroedersstraat 10, B-3000 Leuven, Belgium; 2 Université de Lyon, Université Lyon1, LYON, F-69365, France; 3 INSERM, U851, 21 Avenue Tony Garnier, Lyon, F-69007, France; 4 Hospices Civils de Lyon, Centre National de Référence des légionelles, Faculté de Médecine Laënnec, France

## Abstract

**Background:**

Bacterial genomes harbour a large number of tandem repeats, yet the possible phenotypic effects of those found within the coding region of genes are only beginning to be examined. Evidence exists from other organisms that these repeats can be involved in the evolution of new genes, gene regulation, adaptation, resistance to environmental stresses, and avoidance of the immune system.

**Results:**

In this study, we have investigated the presence and variability in copy number of intragenic tandemly repeated sequences in the genome of *Legionella pneumophila*, the etiological agent of a severe pneumonia known as Legionnaires' disease. Within the genome of the Philadelphia strain, we have identified 26 intragenic tandem repeat sequences using conservative selection criteria. Of these, seven were "polymorphic" in terms of repeat copy number between a large number of *L. pneumophila *serogroup 1 strains. These strains were collected from a wide variety of environments and patients in several geographical regions. Within this panel of strains, all but one of these seven genes exhibited statistically different patterns in repeat copy number between samples from different origins (environmental, clinical, and hot springs).

**Conclusion:**

These results support the hypothesis that intragenic tandem repeats could play a role in virulence and adaptation to different environments. While tandem repeats are an increasingly popular focus of molecular typing studies in prokaryotes, including in *L. pneumophila*, this study is the first examining the difference in tandem repeat distribution as a function of clinical or environmental origin.

## Background

Variable number tandem repeats (VNTRs) are increasingly widely used as molecular markers in prokaryotes. More recently, attention has turned towards examining the possible functional significance of these tandem repeats, especially that of microsatellites, which are often found outside coding regions, [1-7]. Moreover, the biological significance of intragenic tandem repeats, located within coding regions, has been demonstrated for particular genes [8-11]. With the explosion of available bacterial genomic sequences it has become clear that most, if not all, bacterial genomes contain a considerable number of intragenic tandem repeats [12,13]. The finding that intragenic tandem repeats are so widespread in prokaryotes suggests that the few cases documented so far may be only the tip of the iceberg and that some of the many uncharacterized tandem repeats in bacteria possibly play functional roles in pathogenesis and/or in adaptation to environmental stresses.

Here we have investigated the presence and distribution of tandemly repeated sequences in the genome of the opportunistic pathogen, *Legionella pneumophila*, the etiological agent of Legionnaires' disease. This microorganism flourishes naturally in fresh water environments, but is also frequently found in artificial water systems, which are considered the main source of *Legionella *infections in humans [14]. The *L. pneumophila *species comprises over 15 serogroups, of which serogroup 1 is responsible for the majority of human infections. While these Gram-negative bacteria can multiply free-living in culture, it is now widely accepted that intracellular replication in amoebic hosts such as *Acanthamoeba*, *Naegleria*, or *Hartmanella *is essential for the propagation and dissemination of *L. pneumophila *[15]. Industrial settings, altered lifestyle, and a growing number of elderly and immunocompromised individuals have led to an increase in number of reported *L. pneumophila *infections, which occur following inhalation of contaminated aerosols. When *L. pneumophila *reach the lungs, they are ingested by alveolar macrophages wherein they replicate and which they finally destroy. Ultimately host cell lysis and cell death, as well as the extracellular or surface-associated action of bacterial degradative enzymes, result in damage of lung tissue. *L. pneumophila *infections end in an acute and severe, often fatal, pneumonia, if not quickly and correctly diagnosed.

Two recent studies have examined tandem repeats in *L. pneumophila *as a way to discriminate between closely related strains. However, these studies have not differentiated between intergenic and intragenic repeats [16,17]. In addition, researchers studying individual virulence factors in *L. pneumophila *have noted the presence of repeats in these genes, and have suggested possible functional roles for the repeats [18,19]. In this study, we have identified and characterized the tandem repeat variations between 115 strains of *L. pneumophila *serogroup 1, collected from a wide variety of environments and patients.

## Methods

### Origin of bacterial strains and growth conditions

Lab strains 130 b (Klaus Heuner, University of Wurzburg, Germany), Corby (K. Heuner), Lens (Carmen Buchreiser, Institut Pasteur, and CNRS URA, France), and Paris (C. Buchreiser), were obtained from the indicated labs and the Philadelphia strain was obtained from the American Tissue Culture Collection (ATCC). Strains S03, S1, S18, S3, S32, S66, S71, and S88 were isolated via filtration and selective plating from natural water sources collected at different sites throughout Belgium (kind gift of Dr. Priscilla Declerck, Katholieke Universiteit Leuven, Belgium)(unpublished). Strains IMC 1, 5, 16, and 23 were isolated from water taps, air conditioning units, and incubators in a pediatric hospital in Portugal (kind gift of Dr. Milton S. da Costa, University of Coimbra, Portugal) [20]. The 12 "LEA/LE" strains were isolated and serotyped in our laboratory from heat or acid treated swimming water samples (natural and manmade) collected throughout Belgium (kind gift of Dr. Rudy Calders, Provincial Institute for Hygiene, Antwerp, Belgium). The 18 "L" strains were isolated from various manmade environmental water sources (cooling towers, air conditioning units, pipes, irrigation hydrants, showers, water tanks, and fountains) in Spain (kind gift of Dr. Fernando Gonzalez Candelas, University of Valencia, Spain) [21,22]. Strains MGAS-357, MGAS-637, and MGAS-670 were isolated from sputum samples of sporadic clinical infection cases in Belgium (kind gift of Dr. Jan Verhaegen, University Hospital Gasthuisberg, Belgium)(unpublished). Strain HRD-4 was isolated from a sputum samples in Portugal (Dr. Milton S. da Costa)(unpublished). Strains give the "BEL" designation were isolated from sputum samples of clinical infections during three different outbreaks of *Legionella *in Belgium (kind gift of Dr. Marc Struelens, Free University of Brussels, Belgium)(unpublished). Strains ITA-5 and ITA-12 represent two unrelated community-acquired *Legionella *infections from Italy (kind gift of Dr. Isabella Marchesi, University of Modena and Reggio Emilia, Modena, Italy)([23] and unpublished). The 33 "HL" and 6 "LG" strains were isolated from sporadic, epidemic, and endemic patient infections throughout France (National Reference Center on Legionellae, Lyon, France) ([24,25] and unpublished). The 17 "hot springs" strains (ALF, ED, IZ, NMEX, SG) were isolated from boreholes and hot spring runoffs in Portugal and the U.S.A. with a median temperature of 42°C (Dr. Milton S. da Costa)[26,27].

Unless otherwise described, all established *L. pneumophila *strains were grown at 37°C and 5% CO_2 _in buffered yeast extract broth containing α-ketoglutarate (BYE-α) or on buffered charcoal yeast extract broth plates containing α-ketoglutarate (BCYE-α) and supplemented with L-cysteine and ferric pyrophosphate [28]. All strains were stored as glycerol stocks at -80°C and thawed fresh for genomic DNA isolations. For "stress" experiments, cultures of the Philadelphia strain were grown on BCYE-α plates (replated every 3–4 days) at 27°C, 37°C (without CO_2_), 37°C (with CO_2_) and 42°C (without CO_2_) for three months. This work was carried out with permission of the K.U. Leuven Biosafety Council and according to the EU directive 93/88 and 90/219/EC.

### Genomic DNA isolation

Strains were grown overnight in a 5 ml culture of BYE-α. Genomic DNA was isolated from 1 ml of this culture using a Wizard^® ^Genomic DNA Purification Kit (Promega) according to the manufacturer's recommendations. The integrity of the DNA was assessed by agarose gel electrophoresis.

### PCR and sequencing

PCR primers were designed using the Primer3 software [29] and chosen based on regions of high similarity (if possible) between the published Philadelphia, Lens, and Paris strain sequences (see Additional File 1). PCR was performed using SuperTaq DNA polymerase (HT Biotechnology) in 50 μl reaction volumes. Initial denaturation at 94°C for 2 min was followed by 30 cycles of denaturation at 94°C for 30 s, annealing at 45°C for 45 s, and elongation at 72°C for 2 min. The final extension step was 2 min at 72°C. Products were visualized on 1% agarose gels for initial characterization. For sequencing the repeat regions directly, a larger portion of the gene was amplified from each strain and the repeat region was sequenced by VIB Genetic Service Facility, Antwerp, Belgium. Repeats were counted from these sequences using "Tandem Repeats Finder"[30]. Sequence-based typing of all strains except for L430–L2006 was performed using the seven gene method as previously described [31,32]. Strains L430–L2006 were already typed using the older six gene method [21,22].

## Results

### *In silico *screening of the *Legionella pneumophila *genome for intragenic tandemly repeated sequences

The *Legionella pneumophila *serogroup 1 Philadelphia strain [33] published sequence (GenBank accession no. AE017354) was screened for intragenic tandem repeats using the EMBOSS(ETANDEM) software [34]. This resulted in the identification of 85 ORFS containing 95 tandemly repeated sequences. From this list a repeat was considered significant if it met at least one of the following three criteria: the E-tandem score was greater than 50, repeat conservation was greater than 85%, or the size of the repeat was greater than 100 bp and was present in three or more copies. Following this analysis, 39 tandem repeats remained, of which 13 were annotated in the published genome sequence only as "Hypothetical Protein" and discarded from analysis on the basis of the difficulty of assigning putative functions for subsequent analyses and choice as targets for future experiments. The remaining 26 tandem repeats were contained within 23 ORFS and were analyzed further. These 26 repeats ranged in size from 9 bp to 261 bp and were found in copy numbers ranging from 3–19 copies (Table 1). The length of every repeat was divisible by 3, consistent with a strong selective pressure for repeat expansion/deletion to maintain the reading frame.

**Table 1:** Genes containing intragenic tandem repeat arrays in the Philadelphia strain

Gene	Length of repeat (bp)	Copy number	Identity† (%)	Genome annotation‡
LPG0451*	30	18	75.6	IcmE (DotG)
LPG0451*	30	7	72.9	IcmE (DotG)
LPG0688	9	5	88.9	Hsp60, 60 K Heat shock protein
LPG1035	102	3	93.8	Copper efflux ATPase copA2
LPG1038	12	4	89.6	Vrrb
LPG1062	108	8	71.1	TPR repeat protein
LPG1172*	108	8	69.9	TPR repeat protein
LPG1172*	108	4	75.0	TPR repeat protein
LPG1299 (Lpms35)§	18	3	87.0	Transmembrane Tfp pilus assembly protein FimV
LPG1356*	108	4	72.2	TPR repeat protein
LPG1356*	108	3	74.4	TPR repeat protein
LPG1421	261	3	71.4	30S Ribosomal protein S1
LPG1555	21	2	100	Arginine 3rd transport system periplasmic binding protein ArtJ
LPG1602	90	10	65.1	FLJ00180 protein
LPG1948	90	7	75.6	FLJ00180 protein
LPG1958	87	13	74.3	FLJ00180 protein
LPG1976	171	3	73.7	UVB-resistance protein UVR8
LPG2222	108	6	70.7	TPR repeat protein, protein-protein interaction
LPG2224	171	5	73.5	UVB-resistance protein UVR8
LPG2392	87	6	72.2	Leucine rich repeat protein family
LPG2416	105	3	81.9	Ankyrin repeat family protein
LPG2485	102	9	58.5	TPR domain protein
LPG2559	12	4	91.7	ATP-dependant DNA helicase RecG
LPG2639	108	5	65.9	Enhanced entry protein EnhC
LPG2644 (Lpms31)§	45	19	85.4	Tail fiber protein SclB (collagen-like)
LPG2793 (Lpms3)§	96	7	74.0	LepA, interaptin

*Genes containing two distinct repeat arrays. †Percentage identity between repeats. ‡All gene annotations are from the published sequence of *Legionella pneumophila*, Philadelphia strain (GenBank accession no. AE017354). §Annotation used in MLVA typing scheme proposed by [17]. IcmE: intracellular multiplication protein E, DotG: defect in organelle trafficking protein G, Vrrb: variable region with repetitive sequence B, TPR: tetratricopeptide repeat, FimV: fimbriae protein V, Tfp: type IV pili, SclB: *Streptococcus pyogenes *collagen-like protein B, LepA: *Legionella *effector protein A.

### Functional categorization of genes containing tandem repeats

The 23 genes were examined with respect to their subcellular localization using the bacterial protein localization prediction program, PSORTb v.2.0 [35]. These results were compared to the overall subcellular localization predictions of the entire *L. pneumophilia *Philadelphia proteome (Table 2). In general, the genes containing tandem repeats appear to broadly reflect the overall distribution of proteins within a cell, with the exception of a lower proportion of inner membrane proteins, and a higher proportion of periplasmic and extracellular proteins. The increase in the proportion of extracellular proteins is due to the presence of several proteins from the tetratricopeptide repeat (TPR) repeat family (LPG1062, LPG1172, LPG1356, LPG2222) and "enhanced entry protein C" (EnhC, LPG2639) all of which contain multiple copies of a 108 bp repeat (Table 1). This family of "eukaryotic-like" proteins is known to encode both Sel-1 (SLR) and TPR repeat motifs within *L. pneumophilia *[19,36]. Furthermore, these SLR regions are thought to play important roles in protein-protein interactions required for virulence in *Legionella *[19,37], and EnhC has been recently shown to be conserved in virtually every *L. pneumophila *species examined [25] and to play a role in intracellular growth within macrophages [38].

**Table 2:** Subcellular localization of proteins containing tandem repeats

	*L. pneumophila *Philadelphia proteome (n = 2941)	Proteins containing repeats (n = 23)	*Χ*^2 ^p-value
Cytoplasmic	31.1% (n = 915)	21.7% (n = 5)	0.33
Inner Membrane	18.2% (n = 534)	0% (n = 0)	**0.024**
Extracellular	0.8% (n = 22)	21.7% (n = 5)	**< .0001**
Outer Membrane	1.8% (n = 53)	4.3% (n = 1)	0.359
Periplasm	1.2% (n = 36)	8.7% (n = 2)	**0.001**
Unknown	46.9% (n = 1381)	43.4% (n = 10)	0.738

### PCR characterization of repeat variability between strains

In order to determine if a particular repeat was "polymorphic" or "monomorphic", we screened a panel of 47 different *L. pneumophilia *serogroup 1 strains by PCR using primers designed to flank the 26 repeat regions described above. These strains included common lab strains, clinical isolates and environmental isolates (see Methods). Of the 26 repeats, 7 were found to be polymorphic (LPG1038, LPG1299, LPG1555, LPG2224, LPG2416, LPG2644, LPG2793). At the sequence level, the internal conservation of these polymorphic repeats was, on average, higher than that of the monomorphic repeats (unpaired *t*-test, p-value .0021), as observed previously [12]. Genes LPG1299, LPG2644, and LPG2793 correspond to the previously characterized VNTR markers Lpms35, Lpms31, and Lpms3 respectively [17]. Of the genes that were polymorphic, LPG1038, LPG1555, LPG2224, LPG2416, and LPG2793 each possessed only 2 or 3 alleles whereas LPG1299 possessed 22 alleles. The repeat region for gene LPG2644 possesses 12 alleles and more data about LPG2644 and its encoded protein will be described in detail elsewhere (Vandersmissen, L., Coil, D.A., De Buck, E., Lammertyn, E., Anné, J. submitted for publication).

### Patterns of repeat variation

These 7 repeats were further examined in an additional 106 strains, for a total of 153 strains, divided into four strain groups: lab strains (n = 5), environmental strains (n = 59), clinical strains (n = 65), and hot springs strains (n = 24). However, lab strains were not considered for any subsequent statistical analyses. We next performed sequence-based typing on all 153 strains in order to examine the relatedness of the strains. Strains were excluded if they were collected from the same site and possessed both the same SBT type and the same pattern of tandem repeat variation. This resulted in the removal of 38 strains from our analysis, leaving 42 environmental strains, 51 clinical strains, and 17 hot springs strains for a total of 115 strains (see Additional File 2).

The average number of repeats for each gene was calculated across each of the three remaining categories of strains (Table 3). The data was found to have unequal variance between categories (Levene's test), therefore the means were compared using a two-tailed heteroscedastic t-test (Table 3). From a clinical perspective, the most important comparison is between the environmental and clinical samples. Genes LPG1038, LPG1299, LPG2416 and LPG2793 all exhibited significant differences in repeat distribution between clinical and environmental isolates. In all of these cases repeat numbers were higher in the clinical samples than in the environmental samples.

**Table 3:** Comparison of average tandem repeat copy number between strain types

**A**		**Average number of repeats**						
	**Gene**	**1038**	**1299**	**1555**	**2224**	**2416**	**2644**	**2793**
	
	Environmental (E)	2.78	14.52	1.02	4.40	2.04	11.80	6.19
	Clinical (C)	3.5	17.78	1.10	4.71	2.28	12.70	6.45
	Hot springs (H)	3.00	9.65	1.00	5.00	2.41	14.12	7.00
**B**		**Student t-test p-values**						
	
	E versus C	**0.0003**	**0.029**	0.13	0.34	**0.0097**	0.15	**0.044**
	E versus H	0.096	**0.0014**	0.32	0.070	**0.011**	**< .0001**	**< .0001**
	C versus H	**0.0011**	**< .0001**	**0.024**	0.17	0.36	**0.0014**	**< .0001**

*Values in bold were considered significant (p < .05). The mean of each population (Environmental, Clinical, Hot Springs) was compared using a two-tailed heteroscedastic t-test.

### Stability of repeat number

Most tandemly repeated sequences are known to be able to mutate at a faster rate than non-tandemly repeated sequences (for a recent review, see [39]). Therefore, we were interested in examining the stability of these repeats over time, to ensure that our data do not simply represent a "snapshot" in time of repeat copy number in these strains. We began by serially passaging identical cultures of the Philadelphia strain at various temperatures for three months (see Methods). At the end of this period, we measured the repeat copy number in each of the 26 candidate repeat arrays and found that none of them had varied over this time span (data not shown). Because two of the 23 candidate genes are involved in UVB resistance (Table 1) we also examined the stability of these two repeats under repeated exposure to UV light. Plates of Philadelphia strain were exposed to varying lengths (30, 60, 120, 240 seconds) of UVB radiation just after streaking at each passage (~ every 3 days). While there was a noticeable effect on survival of the bacteria, after 10 generations no changes in repeat number were observed for either LPG1976 or LPG2224 (data not shown).

## Discussion

While bacteria in general appear to have a large number of tandem repeats, the possible phenotypic effects of intragenic repeats are only beginning to be examined. Evidence exists from other organisms that variable number tandem repeats are involved in the evolution of new genes, gene regulation, adaptation, resistance to environmental stresses, and avoidance of the immune system. In this work, we have investigated the presence and variability in copy number of tandemly repeated sequences in the genome of *L. pneumophila*, an important human pathogen and model for the study of host-pathogen interactions. We have identified 23 genes containing tandem repeats and determined that seven of them exhibited variability in repeat copy number between strains.

More importantly, we have demonstrated that the distribution of repeat variation is significantly non-random in *L. pneumophila *and therefore may have functional implications. Our results suggest that the number of intragenic tandem repeats found within most genes varies as a function of strain origin. Six of the seven genes examined display distinctive differences between the three groups of strains examined (environmental, clinical, and hot springs). In particular, four genes exhibit significant differences in repeat copy number between environmental and clinical samples. Moreover, for three of the four genes, the distribution of repeat copy number is also significant between clinical samples and hot springs samples, further highlighting the potential significance of the higher repeat copy number found in clinical samples for these genes.

One possible complication with an experiment of this nature is the rate of change of repeat copy number. While a variety of studies have been undertaken examining the mechanism and stability of tandem repeats, none of them addressed intragenic tandem repeats in particular and most have focused on smaller microsatellites (1–6 bp) [39]. In our hands, none of these larger repeats changed in copy number through multiple generations under a variety of conditions. While these conditions do not accurately substitute for a competitive natural environment, these experiments do suggest that intragenic repeat number is reasonably stable over time.

The four genes which exhibit significant differences in repeat copy number between environmental and clinical samples are of particular interest from a clinical perspective since tandem repeats in these genes could play roles in the pathogenesis of *L. pneumophila*. LPG1038 is annotated as "*vrrb*" in the published sequence, however we have been unable to verify any nucleotide or protein homology with the "variable region with repetitive sequence B" (*vrrb*) gene from *Bacillus anthracis *[40]. Furthermore, BLAST searches with this sequence do not produce any significant hits other than those from the published *L. pneumophila *sequences. It is therefore possible that this gene may represent a novel virulence factor in which tandem repeats could play a functional role. LPG1299 is homologous to the *fimV *gene described in *Pseudomonas aeruginosa *[41]. This gene is thought to be involved in twitching motility, possibly through the remodeling of the peptidoglycan layer to enable assembly of type IV pili. Twitching motility is known to be important in the virulence of *P. aeruginosa*, however it has not yet been described in *L. pneumophila*. LPG2416 is a gene of unknown function but contains an ankyrin repeat sequence, thought to mediate protein-protein interactions [42], and which have been recently demonstrated to play a role in the manipulation of *L. pneumophila *host physiology and infection [37,43,44]. Lastly, LPG2793 encodes an effecter of the Icm/Dot type IV secretion system and is known to play a role in the release of *L. pneumophila *from a protozoan host [45,46].

## Conclusion

Overall, our results provide a detailed examination of variable intragenic tandem repeat distribution as a function of strain origin. These data suggest a potential functional role of tandem repeats in adaptation to different environments. Current work is focused on understanding the exact role that intragenic tandem repeats play in particular genes.

## Authors' contributions

DAC helped design the study, carried out the experiments, analyzed the data, and drafted the manuscript. LV participated in the design of the study, the *in silico *analysis, and performed some of the PCR and sequencing. CG and SJ performed the SBT typing of all strains. EL conceived of the study and assisted with the *in silico *analysis. JA participated in the design and coordination of the study and helped to write the manuscript. All authors read and approved the final manuscript.

## Supplementary Material

Additional file 1 **PCR Primers used in this study**. This file lists all of the PCR primers used for tandem repeat amplification in this study. Click here for file

Additional file 2 **Repeat and SBT information for all strains**. This table contains the tandem repeat copy number and SBT type for all 115 strains used in the final analysis. Click here for file
