# Supplementary material for: Intragenic tandem repeat variation between Legionella pneumophila strains
Source: BMC Microbiol. 2008 Dec 10;8:218. doi: 10.1186/1471-2180-8-218 (PMC2639597; doi:10.1186/1471-2180-8-218)
Supplement: Additional file 1 — PCR Primers used in this study. This file lists all of the PCR primers used for tandem repeat amplification in this study. [file 1471-2180-8-218-S1.pdf]

Additional file 1:

Repeat region PCR Primers used in this study

Gene	Direction	Sequence (5' to 3')	Expected fragment size (Philadelphia)
LPG0451	F	GTTGCAGATTGCAGCGTAGA	721bp
LPG0451	R	GAGTAGCGCCAGGAATTGAG	
LPG0451B	F	CGCGTCTGCGATTAGAAAA	297bp
LPG0451B	R	TCCACATCACAACCAGCATT	
LPG0688	F	TATTCTTGATCCAACC	175bp
LPG0688	R	TTACATCATTCCGCC	
LPG1035	F	TGATGTCTATGAATGG	654bp
LPG1035	R	CTTTTGCCGAATCAGG	
LPG1038	F	ACCGTGATGCCAGTTTTGTT	100bp
LPG1038	R	TGGACATAGATGGCATTGGA	
LPG1062	F	CCGCAATTATTATTGGTGTGG	1019bp
LPG1062	R	GAAAGAGGAACCGCCTCAC	
LPG1172	F	TTTATCAGCGCTGGATGTTG	962bp
LPG1172	R	CCAAGTTATTTTGGGCTTCG	
LPG1172B	F	AGATCAAGGATTTGCCGAAG	488bp
LPG1172B	R	ATTTCCACAATTTATTTTTATTCCA	
LPG1299	F	AATCGAAAAAGCTGATGCAGT	186bp
LPG1299	R	TCCATAACGGGCTCAAACAT	
LPG1356	F	TGAAAATGGCGACTACACCA	595bp
LPG1356	R	ATTGGCTGCTTGTGAAAACC	
LPG1356B	F	TGGACATGTCAACGCACAA	300bp
LPG1356B	R	GCGTAAGCTTTAACCAAGCTC	
LPG1421	F	ATTTCCTGGAAGCGAGTCAA	845bp
LPG1421	R	TTTGCTTCAACTTCATCACCA	
LPG1555	F	ATGGCATCTGAACCCTATGC	108bp
LPG1555	R	TTGATCAGGGTTTGCCATAA	
LPG1602	F	GCGCGTTGAACTATTTAATCC	988bp
LPG1602	R	CGATAATTTAATCGTCGCTATATTGG	
LPG1948	F	CAAACAAAAGAAATAATCGCTGAA	682bp
LPG1948	R	TGGTTAATTTACCTTCGATTTGC	
LPG1958	F	CTGTTTCAAATAGGAGAGCACAA	1200bp
LPG1958	R	GCCCATGTTCAAGCTGGTTA	
LPG1976	F	AATATAATCGGCCGCAAATC	661bp
LPG1976	R	TTGTTGATTGCGCTCTTGTC	
LPG2222	F	CACCAGAGGGCTATAAAAAGG	692bp
LPG2222	R	GGCTGCTGTAAACCAAGCAT	
LPG2224	F	ACATCACTTCCATCG	1164bp
LPG2224	R	ATGATGCAATGATTGC	
LPG2392	F	TGGGAGATTTTCACCATGTTT	538bp

LPG2392	R	AAGGAGTGCCAGCTTTTCAA	
LPG2416	F	GTCTGGCCGCAGAGAATG	292bp
LPG2416	R	ATTTCTGCGTAAGCGAGCAT	
LPG2485	F	GCCATTTACCTCAAGCCATT	995bp
LPG2485	R	CATATGCCTGCTGATCACATC	
LPG2559	F	AATCGAACAATGGGGAAGTG	248bp
LPG2559	R	CCCCATTTTGCAGTACTTGG	
LPG2639	F	AACATTGGTATACTGC	840bp
LPG2639	R	CATAACTTACTTTACC	
LPG2644	F	TCACATCACAGATAGC	1299bp
LPG2644	R	TTCCCAGCTCATTACG	
LPG2793	F	ATGAAAGATCATGCAC	1092bp
LPG2793	R	ATCTTCTTCAGATGAG	
